# Decreased lipogenesis-promoting factors in adipose tissue in postmenopausal women with overweight on a Paleolithic-type diet

**DOI:** 10.1007/s00394-017-1558-0

**Published:** 2017-10-26

**Authors:** Caroline Blomquist, Elin Chorell, Mats Ryberg, Caroline Mellberg, Evelina Worrsjö, Elena Makoveichuk, Christel Larsson, Bernt Lindahl, Gunilla Olivecrona, Tommy Olsson

**Affiliations:** 1 Department of Public Health and Clinical Medicine, Medicine, Umeå University, By 6M, M31, SE-901 87 Umeå, Sweden; 2 Department of Public Health and Clinical Medicine, Occupational and Environmental Medicine, Umeå University, Umeå, Sweden; 3 Department of Medical Biosciences, Umeå University, Umeå, Sweden; 4 Department of Food and Nutrition and Sport Science, University of Gothenburg, Gothenburg, Sweden

**Keywords:** Lipoprotein lipase, Obesity, Postmenopausal women, Diet, Fat metabolism

## Abstract

**Purpose:**

We studied effects of diet-induced postmenopausal weight loss on gene expression and activity of proteins involved in lipogenesis and lipolysis in adipose tissue.

**Methods:**

Fifty-eight postmenopausal women with overweight (BMI 32.5 ± 5.5) were randomized to eat an *ad libitum* Paleolithic-type diet (PD) aiming for a high intake of protein and unsaturated fatty acids or a prudent control diet (CD) for 24 months. Anthropometry, plasma adipokines, gene expression of proteins involved in fat metabolism in subcutaneous adipose tissue (SAT) and lipoprotein lipase (LPL) activity and mass in SAT were measured at baseline and after 6 months. LPL mass and activity were also measured after 24 months.

**Results:**

The PD led to improved insulin sensitivity (*P* < 0.01) and decreased circulating triglycerides (*P* < 0.001), lipogenesis-related factors, including LPL mRNA (*P* < 0.05), mass (*P* < 0.01), and activity (*P* < 0.001); as well as gene expressions of *CD36* (*P* < 0.05), fatty acid synthase, *FAS* (*P* < 0.001) and diglyceride acyltransferase 2, *DGAT2* (*P* < 0.001). The LPL activity (*P* < 0.05) and gene expression of *DGAT2* (*P* < 0.05) and *FAS* (*P* < 0.05) were significantly lowered in the PD group versus the CD group at 6 months and the LPL activity (*P* < 0.05) remained significantly lowered in the PD group compared to the CD group at 24 months.

**Conclusions:**

Compared to the CD, the PD led to a more pronounced reduction of lipogenesis-promoting factors in SAT among postmenopausal women with overweight. This could have mediated the favorable metabolic effects of the PD on triglyceride levels and insulin sensitivity.

## Introduction

Obesity, particularly abdominal obesity, is a major cause of morbidity and mortality. Among women, the prevalence of abdominal adiposity increases after menopause and is associated with an increased risk for metabolic disease [1].

Adipose tissue stores energy as triacylglycerols (TGs) in lipid droplets formed through lipogenesis, and fatty acids (FAs) are released from these stored TGs via lipolysis. Both processes are reportedly elevated in insulin-resistant individuals with obesity compared to insulin-sensitive individuals with obesity [2]. The cycle of lipid synthesis and degradation is required for the formation of diacylglycerols (DAGs) and free fatty acids (FFAs), which acts as regulatory ligands of nuclear receptors [3]. Elevated formation of FFAs and DAGs due to increased lipolysis in adipose tissue, may contribute to impaired intracellular insulin signaling, i.e., insulin resistance [2].

TGs in adipose tissue primarily originate from FAs released from TG-rich lipoproteins following lipoprotein lipase (LPL)-mediated intravascular lipolysis [4]. LPL is thus considered a gatekeeper enzyme to play an important role in the initiation and development of obesity [4]. Released FAs can enter adipocytes via either passive diffusion or through diffusion facilitated by the major transport protein CD36 [2].

Within a fat cell, FAs undergo a series of enzymatic reactions leading to their storage as TGs in lipid droplets. The final and likely rate-limiting step in TGs synthesis is catalyzed by diglyceride acyltransferase 2 (DGAT2) [5]. Fatty acid synthase (FAS) is an important factor in *de novo* lipogenesis in adipocytes, and is elevated in cases of obesity and in type 2 diabetes [6]. In cases of obesity, basal lipolysis may be elevated by increased production of pro-inflammatory factors such as TNF-α, increasing transcription of the rate-limiting enzyme adipose triglyceride lipase (ATGL) [7]. Moreover, lipolysis is controlled by a number of lipid droplet-associated proteins that influence droplet formation and stability [5]. In particular, perilipin1 is a key factor that protects TGs from hydrolysis by ATGL [7].

White adipose tissue is not only an energy-storage organ, but also an endocrine organ secreting a variety of adipokines, acting in locally or systemically ways. Adipokines, including leptin, adipsin and adiponectin, have endocrine effects on insulin sensitivity; and leptin also affects energy homeostasis. The secretions of these adipokines are affected by fat storage but the effect of macronutrient content in the diet is not well studied.

A recent study comparing before and after menopause demonstrated that postmenopausal women showed an increased tendency to store TGs in subcutaneous adipose tissue (SAT), associated with increased lipogenesis [8]. Thus, further studies regarding the putative reversibility of altered fat metabolism among postmenopausal women with overweight are of major interest.

We previously made a diet intervention with a 5-week ad libitum Paleolithic type diet (PD), characterized by a moderately increased intake of protein and high contents of monounsaturated fatty acids (MUFAs) and polyunsaturated fatty acids (PUFAs). This diet profoundly decreased abdominal obesity, blood lipid levels, and increased hepatic insulin sensitivity among postmenopausal women with obesity [9]. More recently we made a study on postmenopausal women with obesity, which revealed that a PD had sustained effects on circulating TG levels [10]. Moreover, a PD has also been reported to improve glucose sensitivity, lipid profiles, and blood pressure among healthy sedentary humans without concomitant weight loss [11].

Our hypothesis was that a diet-induced weight loss would affect the levels of adipokines, lipogenesis and lipolysis in postmenopausal women with obesity. We tested in this secondary analysis whether a PD with a high intake of unsaturated fat and a low intake of carbohydrates would have more pronounced beneficial effects on adipokines and key proteins in fat metabolism than a conventional prudent diet with a high carbohydrate content (CD).

## Methods

### Subjects and clinical protocol

A CONSORT flow diagram and additional details regarding inclusion criteria, dietary instructions, and procedures for anthropometry and dual-energy X-ray absorptiometry are described in a previous paper by Mellberg et al. [10].

Briefly, 70 postmenopausal women (age 60.5 ± 5.6 years) with overweight or obesity (BMI, 27–41 kg/m^2^) and normal fasting plasma glucose levels were randomized to an ad libitum Paleolithic-type diet (PD) or a prudent control diet (CD). Only women that had experienced at least 12 consecutive months without menstruation were included in the study. The CD followed the Nordic Nutrition Recommendations aimed to include 15 energy percent (*E*%) protein, 55 *E*% carbohydrates and 30 *E*% fat. The CD was based on high-fiber products, meat, fish, vegetables, fruits and low-fat dairy products. The PD aimed to include 30 *E*% protein, 30 *E*% carbohydrates, and 40 *E*% fat, with recommendations for a high intake of MUFAs and PUFAs, and a relatively low intake of carbohydrate. The PD was based on lean meat, fish, eggs, vegetables, fruits, berries, and nuts. Additional fat sources included avocado and rapeseed and olive oil used in food preparation and dressings. The PD excluded dairy products, cereals, added salt, and refined fats and sugar.

Throughout the entire intervention period, each group participated in a total of 12 group sessions led by dieticians. The group sessions gave information on the intervention diets and how to cook using recipes. They also included group discussions and information regarding dietary impacts on health and behavioral changes. During the first 6 months of the intervention, eight group sessions were held, followed by one group session every 3 months until the end of the intervention.

The present secondary analysis on fat metabolism included 58 women that had abdominal fat biopsies taken at baseline and after 6 months of dietary intervention. Dietary intake was assessed using 4-day (3 week days and 1 weekend day) estimated self-reported food records collected at baseline and monthly for 6 months. The reported food intake was converted to the estimates of energy and nutrient intake using the nutritional analysis package Dietist XP (version 3.0, Kost och Näringsdata AB, Bromma, Sweden) based on the food composition database of the Swedish National Food Administration (2008-03-06) [10].

Physical activity was measured using the Actiheart^®^ monitor during a 7-day period, at baseline and at 6 months, concurrently with the self-reported food records. The study participants gave written informed consent, and the study was approved by the Regional Ethical Review Board at Umeå University. This trial was registered at clinicaltrials.gov as NCT00692536.

Blood samples were obtained after overnight fasting at baseline and 6 months. Plasma glucose and lipid levels were analyzed using a Vitros 5.1FS automated chemistry analyzer (Vitros Slides; Ortho-Clinical Diagnostics, Johnson & Johnson, NJ, USA). FFAs were determined in serum following the ACS-ACOD method using a NEFA-HR kit (Wako, Neuss, Germany). Insulin sensitivity was calculated applying the homeostasis model assessment for insulin resistance (HOMA-IR) [12]. SAT was obtained by needle aspiration under local anesthesia (Xylocaine 10 mg/mL; Astra Zeneca, Södertälje, Sweden), as previously described [13].

### RNA extraction and real-time RT-PCR

Total RNA was extracted from SAT biopsies using the RNeasy^®^ Lipid Tissue Mini kit and the RNA reversed transcribed using TaqMan^®^ reverse transcription reagents as previously described [14]. Relative quantification real-time PCR was performed using an ABI Prism^®^ 7000 Sequence Detection System (Applied Biosystems, Foster City, CA) with Universal PCR Master Mix 2X (Roche Molecular Systems) and TaqMan gene expression assays (Applied Biosystems) for *DGAT2* (Hs01045913_m1), *FAS* (Hs01005622_m1), *LPL* (Hs00173425_m1), ATGL (alias *PNPLA2;* Hs00386101_m1), Perilipin1 (alias *PLIN*; Hs00160173_m1), *CD36* (Hs01567185_m1) and *LRP10* (Hs00204094_m1). Reference genes were evaluated by comparing *PPIA* (Hs999999904_m1) and *LRP10* within the full study cohort using the NormFinder algorithm, and calculated the %CV [15]. Accordingly, *LRP10* appeared to be the most suitable gene. Accordingly, the expression levels of the target genes were normalized to *LRP10*. Due to the limited amounts of adipose tissue in the biopsies we could only analyze gene expression at baseline and after 6 months.

All samples from each subject were analyzed on the same plate in duplicate. To reduce interference from plate biases, subjects were paired and balanced according to diet, fat distribution, insulin sensitivity index, and blood pressure parameters. Samples/subjects were balanced and paired using a space-filling design from a principle component analysis model calculated based on the subjects’ baseline characteristics [16].

### LPL activity and mass measurements

LPL activity and mass were measured in SAT as previously described [17]. The presented data are the mean values of three determinations. For LPL activity, 1 mU corresponds to the release of 1 nmol fatty acids per min. Samples taken at baseline, at 6 months and 24 months from the same individual were analyzed on the same day and in the same assay, to reduce inter-assay variability.

### Analysis of adipokines in serum

Serum concentrations of leptin, adipsin, and adiponectin were determined using the Bio-Rad human diabetes kit (Hercules, CA, USA) following the manufacturer’s instructions, with the addition that all samples were centrifuged for 30 s at 11,000×*g* to remove any debris. All samples were assayed in duplicate and analyzed using the Luminex 200 Labmap system (Austin, TX, USA). Data were analyzed using Bio-Plex Manager software version 4.1.1 or 6.0 (Bio-Rad). Protein concentrations were interpolated from the appropriate standard curve. Mean %CV values were 4.0% for adiponectin, and 7.8% for adipsin and leptin.

### Statistical analysis

#### PCA/OPLS

We performed further sample comparison modeling using a multivariate data analysis strategy to elucidate intervention-related effects on the whole fat metabolism profile. First, the data were inspected using principal components analysis (PCA) to detect potential outliers and clusters. Second, each individual’s sample collected after 6 months of intervention was subtracted from its baseline sample and missing data excluded. At last, we applied a variant of orthogonal partial least squares analysis (OPLS) [18], OPLS-effect projections (OPLS-EP) [19]. OPLS-EP extracts metabolic profiles based on paired analyses of individual effects, i.e., the dietary intervention effect. Because each subject acted as her own control, this strategy minimizes the influence from confounding factors, such as inter-individual variation [19]. The multivariate models were validated by calculating *P* values based on ANOVA from the cross-validated scores (CV-ANOVA). To ensure proper cross-validation groups and reduce the chance of creating an over-fitted model, special consideration was taken to keep the same participants in the same group. The multivariate confidence intervals presented here were based on jack-knifing [20]. OPLS-EP analyses were performed during weight loss (0–6 months) and weight maintenance (6–24 months).

#### Generalized estimating equations

General estimating equations (GEE) and linear regression analyses were performed using IBM SPSS Statistics for Mac, Version 22.0 (IBM Corp., Armonk, NY, USA). Data describing the anthropometrical and biochemical parameters are presented as mean ± SD. The sample size was estimated using power analysis based on changes in fat mass in a pilot study of postmenopausal women on a PD. It was estimated that 30 participants were needed in each group to achieve *P* < 0.05 with 80% power. The effects of diet over time were analyzed using separate multiple regression models, each including diet group, time, and the group-by-time interaction as predictors. Regression parameters were estimated using GEE, a method that tolerates some degree of between-group variance. An exchangeable correlation structure was used to model the dependence between repeated measurements within participants. Prior to analysis, dependent variables with a skewed distribution were transformed using natural logarithms. Outcome is presented as *P* values for the included factors and estimated marginal means, with corresponding 95% confidence intervals for each diet at each time-point. *P* value of < 0.05 was considered statistically significant.

#### Linear regression analysis

Univariate linear regression analyses were used to identify and characterize the relationship between a dependent variable and an independent variable. Outcome was presented using *P* values for the included factor and the coefficient of correlation R.

## Results

A previous publication describes the results regarding anthropometry and metabolic functions, including circulating lipid levels and insulin sensitivity in all 70 participants [10]. This secondary analysis presents lipogenesis and lipolysis in adipose tissue in the 58 women with fat biopsies during the first 6 months of the study period. LPL mass and activity were analyzed at 24 months. Gene expression was not analyzed due to lack of fat tissue.

### OPLS

**Fig. 1:**
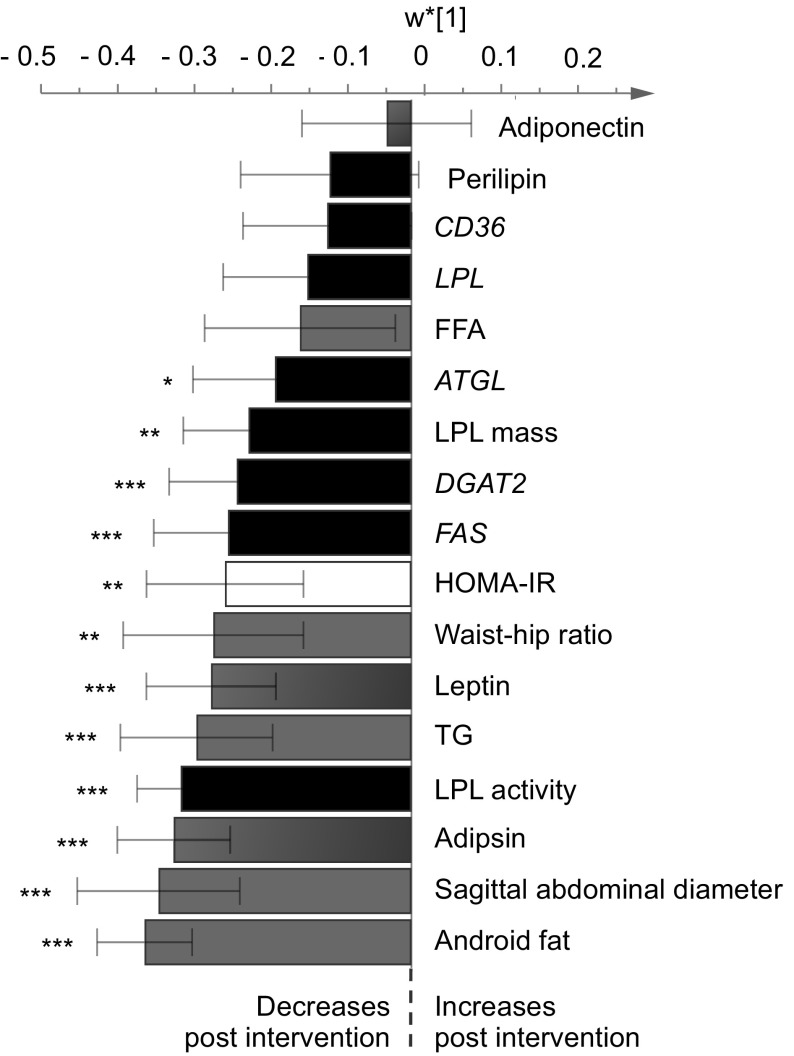
A multivariate model of individual differences in postmenopausal women with overweight comparing samplings at baseline to those after 6 months of an ad libitum Paleolithic type diet (PD) intervention. Variables with negative axis values (multivariate OPLS loadings, w* [1]) are decreased after the intervention and vice versa for those with positive axis values. The shown confidence intervals are multivariate confidence interval, based on jack-knifing using a 95% confidence level. Bars labeled with stars are significantly altered after the intervention by means of two-sided paired* t*-tests, i.e. *P < 0.05, **P < 0.01, ***P  < 0.001. *ATGL* adipose triglyceride lipase, *DGAT2* diglyceride acyltransferase 2, *FFAs* free fatty acids, *FAS* fatty acid synthase, *LPL* lipoprotein lipase; *TGs* triacylglycerols

The OPLS analysis included the following variables: Fat distribution, blood lipids, insulin sensitivity, gene expression and activity levels of key proteins involved in lipogenesis and lipolysis, and adipokines (Fig. 1), at 6 months. We obtained a significant OPLS model only for the PD group (Fig. 1). However, an additional analysis including both groups in the same OPLS model revealed identical patterns of the included variables for both diets as for the PD group alone (data not shown). This suggests that the PD group response on the included variables is more pronounced as compared to the CD group and that no new information is to be found when including both groups in the same model.

Sagittal abdominal diameter, android fat mass, and plasma TG levels decreased significantly, with android fat mass showing the most pronounced reduction. Insulin resistance (estimated by the HOMA-IR index) decreased significantly, with concomitant reductions of adipsin and leptin. With regards to lipogenesis and lipolysis, LPL activity showed the most pronounced reduction, followed by *FAS, DGAT2*, and LPL mass. We also detected a reduced expression of the *ATGL* gene, a key factor in intracellular lipolysis.

### Generalized estimating equations

#### Anthropometric data

After 6 months of the intervention the PD group showed significantly larger reductions in body weight and sagittal abdominal diameter compared to the CD group (Table 1).

**Table 1:** Changes of anthropometric data, serum lipids and adipokines in postmenopausal women with overweight at baseline and after 6 months of an intervention with an ad libitum Paleolithic-type diet (PD) or prudent control diet (CD)

	PD	Changes 0–6 months		CD	Changes 0–6 months		Model effect Diet by time
	Baseline	6 months	*P*	Baseline	6 months	*P*	*P*
Age (years)	60 ± 5.5			62 ± 5.7			
Weight (kg)	87 ± 10	− 7.8 ± 4.5	<0.001	87 ± 9.6	− 3.9 ± 4.6	< 0.001	< 0.05
Sagittal abdominal diameter (cm)	22 ± 2.1	− 4.3 ± 3.3	<0.001	22 ± 2.1	− 0.03 ± 1.9	< 0.001	< 0.001
Waist/hip	0.93 ± 0.07	− 0.05 ± 0.07	<0.001	0.94 ± 0.06	− 2.4 ± 0.04	< 0.001	NS
Serum FFAs (mmol/L)	0.49 ± 0.20	− 0.06 ± 0.19	NS	0.52 ± 0.17	− 0.06 ± 0.14	< 0.05	NS
Serum TGs (mmol/L)	1.2 ± 0.53	− 0.39 ± 0.41	<0.001	1.3 ± 0.55	− 0.11 ± 0.38	NS	< 0.001
Cholesterol in serum (mmol/L)	5.9 ± 0.81	− 0.66 ± 0.74	< 0.001	5.6 ± 1.2	− 0.38 ± 0.82	0.035	NS
LDL-C (mmol/L)	3.9 ± 0.76	− 0.43 ± 0.58	< 0.001	3.7 ± 1.1	− 0.30 ± 0.63	0.037	NS
HDL-C (mmol/L)	1.5 ± 0.36	− 0.07 ± 0.30	NS	1.3 ± 0.24	− 0.04 ± 0.20	NS	NS
HOMA-IR	1.8 ± 1.1	− 0.32 ± 1.3	< 0.01	2.2 ± 1.0	− 0.08 ± 1.3	NS	NS
Adiponectin (mg/L)	38 ± 13	− 3.7 ± 7.0	NS	37 ± 12	− 5.6 ± 8.1	< 0.05	NS
Leptin (ng/L)	12 ± 5.0	− 4.3 ± 5.1	< 0.001	11 ± 4.4	− 2.7 ± 2.7	< 0.001	NS
Adipsin (ng/L)	340 ± 110	− 100 ± 76	< 0.001	330 ± 120	− 110 ± 106	< 0.001	NS

Data are shown as mean ± SD. *n* = 23–25 for the CD grou*p; n* = 32–33 for the PD group. Different *n* within a group is due to missing samples and different *n* between groups is due to a higher dropout rate in the CD group. Regression parameters were estimated by generalized estimating equations

*FFAs* free fatty acids, *HDL-C* high-density lipoproteins cholesterol, *LDL-C* low-density lipoprotein cholesterol, *TGs* triacylglycerols

#### Reported energy intake

The reported energy intake decreased similarly in both groups, while physical activity levels remained stable (Table 2). The reported intake of protein increased significantly more in the PD group compared to the CD group, but did not reach the target level of 30 *E*%. The PD group also reported a significantly higher intake of unsaturated FAs and cholesterol than the CD group (Table 2). Compared to baseline, the PD group reported a significantly decreased intake of carbohydrates, which was significantly lower than that reported by the CD group (Table 2). The intake ratio of fiber-to-carbohydrate increased in both groups and was more pronounced in the PD group compared to the CD group (Table 2). The reported intake of monosaccharides and disaccharides remained stable over time in both groups.

**Table 2:** Changes of nutrient intake and physical activity in postmenopausal women with overweight at baseline and at 6 months of an intervention with an ad libitum Paleolithic-type diet (PD) or prudent control diet (CD)

	PD			CD			Model effect diet by time
	Baseline	Change 0–6 months	*P*	Baseline	Change 0–6 months	*P*	*P*
Energy intake (MJ/d)	8.4 ± 1.5	− 1.5 ± 1.5	< 0.001	8.7 ± 1.6	− 1.9 ± 1.5	< 0.001	NS
PAEE (MJ/d)	3.2 ± 0.82	− 0.10 ± 0.82	NS	3.3 ± 1.1	− 0.08 ± 0.90	NS	NS
Carbohydrate intake (*E*%)	46 ± 4.1	− 17.0 ± 5.7	< 0.001	46 ± 4.5	− 1.5 ± 5.9	NS	< 0.001
Mono- and disaccharide intake (E%)	18 ± 5.7	0.73 ± 5.2	NS	20 ± 6.7	− 1.2 ± 7.3	NS	NS
Fiber intake (g)/carbohydrate intake (g)	0.11 ± 0.02	0.09 ± 0.03	< 0.001	0.10 ± 0.02	0.03 ± 0.03	< 0.001	< 0.001
Fat intake (*E*%)	34 ± 3.6	10 ± 6.7	< 0.001	34 ± 3.8	− 2.5 ± 4.8	< 0.01	< 0.001
SFAs intake (*E*%)	13 ± 2.1	− 3.0 ± 3.2	< 0.001	13 ± 2.0	1.8 ± 2.5	< 0.001	NS
MUFAs intake (*E*%)	13 ± 1.9	7.9 ± 4.0	< 0.001	13 ± 2.1	− 1.3 ± 2.4	< 0.01	< 0.001
PUFAs intake (*E*%)	5.5 ± 1.3	4.2 ± 3.0	< 0.001	5.4 ± 1.1	0.21 ± 1.8	NS	< 0.001
Cholesterol intake (*E*%)	0.13 ± 0.03	0.29 ± 0.56	< 0.001	0.15 ± 0.04	0.01 ± 0.06	NS	< 0.001
Protein intake (*E*%)	17 ± 1.9	6.3 ± 2.9	< 0.001	17 ± 2.5	1.9 ± 2.7	< 0.001	< 0.001

Data are presented as mean ± SD. *n* = 23–25 for the CD group; *n* = 32–33 for the PD group. Different *n* within a group is due to missing samples and different *n* between groups is due to a higher dropout rate in the CD group. The regression parameters were estimated by generalized estimating equations

*MUFAs* monounsaturated fatty acids, *PAEE* physical activity energy expenditure, *PUFAs* polyunsaturated fatty acids, *SFAs* saturated fatty acids

#### Circulating lipids and HOMA-IR

Serum TGs decreased significantly more in the PD group compared to the CD group (Table 1). Total serum cholesterol levels and LDL-cholesterol (LDL-C) decreased in both groups, without differences between groups (Table 1). The levels of HDL cholesterol and FFA remained stable in both groups (Table 1). The HOMA-IR index decreased significantly in the PD group, without significant difference between diet groups (Table 1).

#### Lipogenesis- and lipolysis promoting factors

**Fig. 2:**
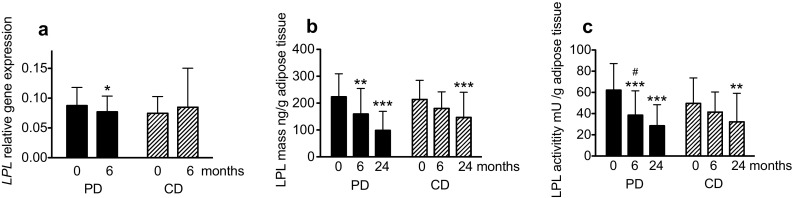
Lipoprotein lipase (LPL) relative gene expression (**a**), mass (**b**), and enzyme activity (**c**) in postmenopausal women with overweight after a 6-month and 24 month intervention with an ad libitum Paleolithic type diet (PD) or prudent control diet (CD) compared with at baseline. Data are presented as mean ± SD. *n* = 23–25 for the CD group; *n* = 32–33 for the PD group. Different *n* within a group is due to missing samples and different *n* between groups is due to a higher dropout rate in the CD group. The regression parameters were estimated using generalized estimating equations. Difference from baseline: *P < 0.05, **P < 0.01, ***P < 0.001; difference in change between groups (diet × time effect) ^#^ *P* < 0.05

**Fig. 3:**
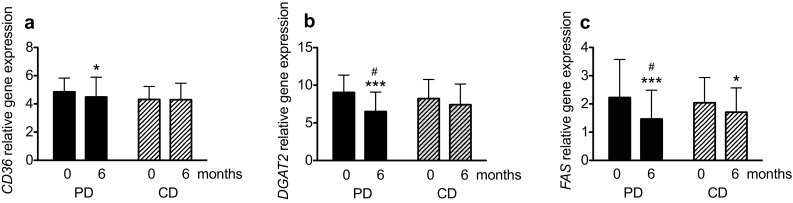
Relative expressions of *CD36* (**a**), *DGAT2* (**b**) and *FAS* (**c**), that encode proteins involved in lipogenesis in postmenopausal women with overweight after a 6-month intervention with an ad libitum Paleolithic-type diet (PD) or prudent control diet (CD) compared with at baseline. Data are presented as mean ± SD. *n* = 23–25 for the CD group; *n* = 32–33 for the PD group. Within-group differences in *n* were due to missing samples and between-group differences in *n* were due to a higher dropout rate in the CD group. The regression parameters were estimated using generalized estimating equations. Differences from baseline: *P < 0.05, **P < 0.01, ***P < 0.001; difference in change between groups (diet x time effect) ^#^ *P* < 0.05. *DGAT2* diglyceride acyltransferase 2, *FAS* fatty acid synthase

**Fig. 4:**
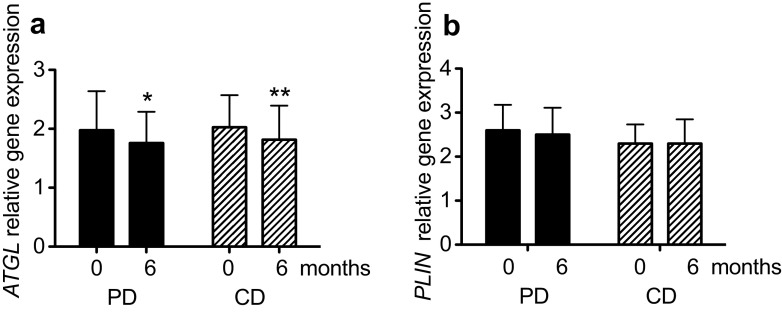
Relative expressions of *ATGL* (**a**) and *PLIN* (**b**), that encode proteins involved in lipolysis in postmenopausal women with overweight after a 6-month intervention with an *ad libitum* Paleolithic-type diet (PD) or prudent control diet (CD) compared with at baseline. Data are presented as mean ± SD. *n* = 23–25 for the CD group; *n* = 32–33 for the PD group. Within-group differences in *n* were due to missing samples and between-group differences in *n* were due to a higher dropout rate in the CD group. The regression parameters were estimated using generalized estimating equations. Differences from baseline: *P < 0.05, **P < 0.01, ***P < 0.001. *ATGL* adipose triglyceride lipase, *PLIN* perilipin1

The gene expressions of *LPL* and *CD36* were significantly decreased in the PD group, but no between-group differences were found (Figs. 2a, 3a). Expressions of *DGAT2* and *FAS* decreased significantly more in the PD group compared to the CD group (*P* < 0.05 for both; Fig. 3b, c). The expression of *ATGL* decreased significantly in both groups, with no differences between groups (Fig. 4a). Perilipin1 mRNA levels were unchanged in both diet groups during the intervention (Fig. 4b).

LPL mass, and activity levels decreased significantly in the PD group after 6 and 24 months, and there were significant differences in changes of LPL activity between the diet groups at both 6 and 24 months (*P* < 0.05 for both; Fig. 2b, c). Significant associations were found between the changed LPL activity and LPL mass at 24 months using linear regression analyses for both the PD group (*R* = 0.73, *P* = 0.001) and the CD group (*R* = 0.61, *P* = 0.019).

#### Adipokines in serum

Serum levels of leptin and adipsin decreased in both groups with no significant differences between groups (Table 1). Adiponectin levels decreased in the CD group, without differences between groups (Table 1).

### Linear regression analysis

Using linear regression analyses, we tested possible associations between the main outcomes related to adipose tissue fat metabolism (i.e., *LPL, DGAT2, FAS, CD36* and *ATGL* gene expressions; LPL mass and activity) and sagittal abdominal diameter, diet intake (i.e., carbohydrates and PUFAs) or HOMA-IR/insulin.

In the PD group, a significant association at 24 months was found between changes in sagittal abdominal diameter and LPL activity (*R* = 0.47, *P* < 0.05) and at 6 months between sagittal abdominal diameter and gen expression of *DGAT2* (*R* = 0.48, *P* < 0.001), *FAS *(*R* = 0.48, *P* < 0.05), *CD36* (*R* = 0.25, *P* < 0.05), LPL mass (*R* = 0.36, *P* < 0.05) and LPL activity (*R* = 0.58, *P* < 0.001). There were also significant associations between reported intake of carbohydrate and gene expression of *DGAT2* (*R* = 0.38, *P* < 0.01), *FAS* (*R* = 0.27, *P* < 0.05), *CD36* (*R* = 0.25, *P* < 0.05) and for LPL mass (*R* = 0.33, *P* < 0.05) and LPL activity (*R* = 0.44, *P* < 0.01) at 6 months in the PD group. Furthermore, significant associations at 6 months were found in the PD group between reported intake of PUFA and gene expression of *DGAT*2 (*R* = 0.42, *P* < 0.001), *FAS* (*R* = 0.34, *P* < 0.01), and for LPL activity (*R* = 0.34, *P* < 0.05). Significant associations between LPL mass and HOMA-IR (*R* = 0.38, *P* < 0.05) and circulating insulin levels (*R* = 0.42, *P* < 0.05) were found at 6 months in the PD group. No significant associations were found in the CD group.

## Discussion

Previous studies have shown that an ad libitum PD with a high content of MUFA and PUFA, a relatively low intake of carbohydrate, and a high fiber-to-carbohydrate ratio can have major positive effects on metabolic balance, including increased glucose tolerance and decreased serum TGs [9, 10, 21, 22]. Our findings suggest that these beneficial effects of the PD can be partly mediated by decreased levels of lipogenesis-promoting factors in SAT. The OPLS analysis revealed that the most pronounced effects were on fat distribution factors, circulating TG levels, and adipose LPL activity within the PD group during the first 6 months of intervention.

The greatly reduced LPL activity in SAT  in the PD group was paralleled by a decreased LPL mass. The trend was similar in the CD group, but less pronounced compared to the PD group. Earlier studies of the impact of weight loss on LPL activity in SAT show discordant results [23, 24]. In support of our present data, prior studies have shown reduced adipose tissue LPL activity after surgically induced weight loss in individuals with morbidly obesity [25–27]. Additionally, a study of postmenopausal women with overweight or obesity after a 6-month hypocaloric dietary intervention demonstrated that decreased LPL activity was associated with reductions in abdominal adiposity, total cholesterol, LDLs and TGs [28]. Accordingly, the PD group in the present study showed a more pronounced decrease in LPL activity, associated with greater reductions of abdominal adiposity compared to the CD group. The association between changes of LPL activity and sagittal abdominal diameter in the PD group was verified by both the linear regression analysis and multivariate analysis via OPLS. LPL in adipose tissue is predominantly regulated at the post-translational level by nutritional factors, such as fasting, glucose and insulin [4, 29, 30]. Insulin has effects also at the level of *LPL* gene transcription [31]. The significant decrease in LPL protein mass may be a consequence of the lower intake of carbohydrate and circulating insulin, which were significantly associated to LPL mass in the PD group at 6 months.

The PD group also showed a significantly decreased expression of *CD36*, which could reduce FA uptake and utilization [32]. The expression of *CD36* is upregulated by insulin, and the expression in adipose tissue is also upregulated in obesity and in type 2 diabetes patients [33]. Genetic studies have revealed that variations within the *CD36* locus are associated with metabolic dysfunction through effects on whole-body adiposity [34]. From a metabolic perspective, partial *CD36* deficiency is associated with a beneficial phenotype, such that the *CD36* reduction in the PD group was associated with decreased abdominal adiposity at 6 months in the PD group [35]. Furthermore, the expression of the important lipogenic enzyme *DGAT2* decreased significantly more in the PD group compared to the CD group. Gene expression of *DGAT2* is downregulated in adipocytes by weight reduction in humans and is regulated by nutritional factors such as glucose and PUFAs [36–38]. Carbohydrate and PUFA intake were associated with *DGAT2* gene expression in the PD group at 6 months and may explain the more pronounced decreased *DGAT2* expression found in this study group.

Both diet groups showed decreased expression of the lipogenic enzyme FAS. De novo lipogenesis through *FAS* in the liver is upregulated by glucose, fructose and insulin, and are downregulated by a high-fat diet and possibly by PUFAs [6]. The expression of *FAS* in the adipose tissue was more strongly influenced in the PD group than in the CD group, possibly due to the reduced carbohydrate content and/or increased PUFA content in the PD, and/or by the decreased circulating insulin levels in the PD group [6]. This is supported by the association between intake of PUFA and carbohydrates to *FAS* expression at 6 months in the PD group. Notably, the regulatory responsiveness of *FAS* in adipose tissue is less pronounced than in liver [6].

Both diet groups showed decreased expression of the *ATGL* gene, indicating reduced intracellular lipolysis of stored TGs. This finding is consistent with earlier weight-loss studies in humans with obesity following a hypocaloric diet, suggesting that weight loss per se determines this reduction of gene expression [39, 40]. Activation and recruitment of lipases such as ATGL to the lipid droplet surface are regulated by perilipin 1 [41]. We did not find any changes in perilipin 1 gene expression. The explanation for this unaltered expression may be regulation on the protein level by phosphorylation. Importantly, an overall reduction of basal lipolysis in adipose tissue may lower the risks of ectopic fat storage and reduced insulin sensitivity in other tissues, such as skeletal muscle [42].

The PD group showed an improved metabolic situation manifested by increased insulin sensitivity, as indicated by a decreased HOMA-IR index, and lower circulating leptin and adipsin levels. Leptin represses food intake and increases energy expenditure, and is elevated in women with a metabolic syndrome, likely due to leptin resistance or “hypothalamic leptin insufficiency” [43, 44]. Increased levels of adipsin are reported in postmenopausal women with obesity, and have been suggested to be important for the development of a metabolic syndrome in this patient group [43]. While earlier studies have reported stable or increased adiponectin levels during weight reduction in postmenopausal women [45, 46], the CD group in our present study showed a significant decrease in serum adiponectin levels. The differences between groups may be due to different intake of FFAs that may affect PPARγ, an obligatory transcription factor for adiponectin. Notably, we analyzed total adiponectin levels in blood, while the high-molecular-weight form of adiponectin is considered the most etiologically important component with regards to metabolic effects [47].

### Strengths and limitations

The strengths of this study include a relatively low total dropout rate and the relatively long intervention time. The higher dropout rate in the CD group, largely due to lack of motivation, may have influenced the between-group differences in weight reduction and body composition. Since LPL activity and mass was expressed per g adipose tissue, and the fat cell size is expected to decrease on weight loss due to the reduced content of TG, we may have underestimated the reduction of LPL in adipose tissue. There was no energy restriction but the energy intake decreased in both groups equally at 6 months. A constant energy intake is preferable when evaluating the impact of the macronutrient change on metabolism. Finally, the present study participants were relatively healthy. Future interventions should include subjects with different degrees of metabolic dysfunction, and also include measurements of fat cell size.

## Conclusion

Our present results show that a PD, high in PUFAs and low in carbohydrates, has a more pronounced effect on adipose tissue lipid metabolism than a CD by reducing gene expression of *DGAT2* and *FAS* at 6 months and decreasing LPL activity at 24 months despite similar weight loss. This is linked to improved insulin sensitivity at 6 months and a more pronounced reduction of circulating TGs, suggesting that a PD may be a promising tool to decrease cardiovascular risk in healthy postmenopausal women with overweight.
